# VZV-specific T-cell levels in patients with rheumatic diseases are reduced and differentially influenced by antirheumatic drugs

**DOI:** 10.1186/s13075-018-1742-5

**Published:** 2018-11-09

**Authors:** David Schub, Gunter Assmann, Urban Sester, Martina Sester, Tina Schmidt

**Affiliations:** 1 Department of Transplant and Infection Immunology, Saarland University, 66421 Homburg, Germany; 2 Department of Internal Medicine I, Saarland University, Homburg, Germany; 3 Department of Internal Medicine IV, Saarland University, Homburg, Germany

**Keywords:** T cells, Varicella zoster virus, bDMARDs, Antirheumatic medication, Rheumatic patients

## Abstract

**Background:**

Varicella zoster virus (VZV)-specific cellular immunity is essential for viral control, and the incidence of VZV reactivation is increased in patients with rheumatic diseases. Because knowledge of the influence of antirheumatic drugs on specific cellular immunity is limited, we analyzed VZV-specific T cells in patients with rheumatoid arthritis (RA) and seronegative spondylarthritis (SpA), and we assessed how their levels and functionality were impacted by disease-modifying antirheumatic drugs (DMARDs). A polyclonal stimulation was carried out to analyze effects on general effector T cells.

**Methods:**

CD4 T cells in 98 blood samples of patients with RA (*n* = 78) or SpA (*n* = 20) were quantified by flow cytometry after stimulation with VZV antigen and the polyclonal stimulus *Staphylococcus aureus* enterotoxin B (SEB), and they were characterized for expression of cytokines (interferon-γ, tumor necrosis factor [TNF]-α, interleukin [IL]-2) and markers for activation (CD69), differentiation (CD127), or functional anergy programmed death 1 molecule [PD-1], cytotoxic T-lymphocyte antigen 4 [CTLA-4]. Results of patients with RA were stratified into subgroups receiving different antirheumatic drugs and compared with samples of 39 healthy control subjects. Moreover, direct effects of biological DMARDs on cytokine expression and proliferation of specific T cells were analyzed in vitro.

**Results:**

Unlike patients with SpA, patients with RA showed significantly lower percentages of VZV-specific CD4 T cells (median 0.03%, IQR 0.05%) than control subjects (median 0.09%, IQR 0.16%; *p* < 0.001). Likewise, SEB-reactive CD4 T-cell levels were lower in patients (median 2.35%, IQR 2.85%) than in control subjects (median 3.96%, IQR 4.38%; *p* < 0.05); however, expression of cytokines and cell surface markers of VZV-specific T cells did not differ in patients and control subjects, whereas SEB-reactive effector T cells of patients showed signs of functional impairment. Among antirheumatic drugs, biological DMARDs had the most pronounced impact on cellular immunity. Specifically, VZV-specific CD4 T-cell levels were significantly reduced in patients receiving TNF-α antagonists or IL-6 receptor-blocking therapy (*p* < 0.05 and *p* < 0.01, respectively), whereas SEB-reactive T-cell levels were reduced in patients receiving B-cell-depleting or IL-6 receptor-blocking drugs (both *p* < 0.05).

**Conclusions:**

Despite absence of clinical symptoms, patients with RA showed signs of impaired cellular immunity that affected both VZV-specific and general effector T cells. Strongest effects on cellular immunity were observed in patients treated with biological DMARDs. These findings may contribute to the increased susceptibility of patients with RA to VZV reactivation.

**Electronic supplementary material:**

The online version of this article (10.1186/s13075-018-1742-5) contains supplementary material, which is available to authorized users.

## Background

The varicella zoster virus (VZV) establishes lifelong persistence, thereby requiring permanent control by the host immune system. As a consequence, viral reactivation, mainly presenting as herpes zoster with a median incidence of 4–4.5 per 1000 person-years in the general population [1], preferentially occurs in individuals with impaired immune function. Large observational studies demonstrated a higher rate of herpes zoster in patients with rheumatic disease [2–4]; comparison between different autoimmune diseases revealed the highest age-standardized incidence of herpes zoster in patients with systemic lupus erythematosus (SLE), followed by inflammatory bowel disease and rheumatoid arthritis (RA) [4].

During the last several years, cellular immunity was identified as a main contributor to efficient control of VZV in both immunocompetent and immunocompromised persons [5–8]. This also holds true for patients with autoimmune diseases, where VZV-specific CD4 T cells seem crucial to preventing reactivation [8, 9]. We have previously found in immunocompetent individuals and various groups of immunocompromised patients with and without herpes zoster that VZV-specific CD4 T cells show distinct changes in phenotype and functionality in association with herpes zoster [10]. Interestingly, when compared with control subjects, first evidence suggests that patients with rheumatic diseases showed lower levels of VZV-specific CD4 T cells and an increased expression of the inhibitory molecule cytotoxic T-lymphocyte antigen 4 (CTLA-4) on polyclonally stimulated effector T cells, even in the absence of acute VZV reactivation [10]. This indicates a general impairment of cellular immunity that may be a consequence of the autoimmune disease itself or be caused at least in part by treatment with immunomodulatory antirheumatic drugs such as conventional and biological disease-modifying antirheumatic drugs (cDMARDs and bDMARDs, respectively). Although these immunological alterations may predispose for an increased zoster incidence in patients with rheumatic diseases, evidence on the association of different treatment modalities and the risk for herpes zoster is conflicting (reviewed in [11, 12]). In addition, studies on the influence of antirheumatic medication on VZV-specific cellular immunity are limited. Therefore, the aim of the present study was to analyze the impact of disease entity and antirheumatic medication on VZV-specific and general effector T-cell immunity in patients with RA and seronegative spondylarthritis (SpA). Furthermore, the effect of different antirheumatic drugs on effector T cells was analyzed in vitro.

## Methods

### Recruitment of the study population

Patients with rheumatic diseases, including RA and different types of seronegative spondylarthritis (SpA, including psoriatic arthritis [PsA] and ankylosing spondylitis [AS]), were recruited. Seven patients with RA had more than one blood sample analyzed because their antirheumatic medication was changed at least once during the period of patient enrollment. Because VZV-specific T-cell levels and phenotype are altered in patients with herpes zoster and were shown to normalize within 3 months after resolution [10], patients who had experienced active herpes zoster within the last 3 months before sample acquisition were excluded from major analyses. Likewise, VZV-immunoglobulin G (IgG)-seronegative individuals were excluded. Healthy immunocompetent individuals were recruited as control subjects. Patients had received treatment with cDMARDs or bDMARDs for at least 12 weeks to ensure sufficient exposure to the antirheumatic agent. Patients who did not receive any antirheumatic therapy (except steroids) for at least 12 weeks before blood sampling were termed “therapy-naïve.” If steroids were given, the dosage ranged from 1.5 mg to 40 mg prednisolone equivalent daily (mean 6.4 ± 7.4 mg/d). Additional information regarding history of previous episodes of herpes zoster was collected from the study participants and the treating physician.

### Quantification and characterization of VZV-specific and SEB-reactive T cells

T cells from heparinized whole blood were stimulated in vitro and incubated for 6 h exactly as described before [10]. In brief, blood samples were stimulated with a lysate of VZV-infected fibroblasts, uninfected control lysate (negative control; Virion/Serion, Würzburg, Germany), and 2.5 μg/ml *Staphylococcus aureus* enterotoxin B (SEB) (positive control; Sigma-Aldrich, St. Louis, MO, USA), respectively. All stimulations were performed in the presence of 1 μg/ml anti-CD28 and anti-CD49d (BD Biosciences, San Jose, CA, USA). The last 4 h of stimulation was carried out in the presence of 10 mg/ml brefeldin A. Thereafter, cells were fixed and immunostained with antibodies toward CD4, CD69, interferon (IFN)-γ, interleukin (IL)-2, tumor necrosis factor (TNF)-α, CTLA-4, the programmed death 1 molecule (PD-1) (all from BD Biosciences), and CD127 (eBioscience, San Diego, CA, USA). Flow cytometric analyses were performed on a FACSCanto II using FACSDiva version 6.1.3 software (BD Biosciences). Percentages of VZV-specific CD4 T cells were calculated by subtracting the results obtained after VZV-specific stimulation by those of the negative control. The experimental approach including the detection limit of 0.02% VZV-specific CD4 T cells was established before [10]. Based on serology as a gold standard, this assay has a sensitivity of 92% and a specificity of 74% [10], and the stimuli are able to detect VZV-specific T cells in both infected individuals [10] and after varicella vaccination (Additional file 1: Figure S1).

For analysis of late cytokine expression and proliferation, blood samples were processed as described above, but incubation time was prolonged to 36 h. Proliferation was assessed as described before [13] by incorporation of 500 mM bromodeoxyuridine (BrdU) (Sigma-Aldrich) that was added after 28 h. After fixation, cells were stained with antibodies toward CD4, CD8, CD69, IFN-γ, and BrdU (all from BD Biosciences).

### Preincubation of immune cells with antirheumatic and other immunosuppressive agents

Whole blood (300 μl) was preincubated at 37 °C, 5% CO_2_, for 4 h with estimated maximum plasma levels of different antirheumatic and other immunosuppressive agents as well as with fivefold lower and fivefold higher concentrations (tenfold for methylprednisolone [MP]), respectively. Estimated maximum plasma levels were 150 μg/ml for abatacept, 100 μg/ml for adalimumab, 2.5 μg/ml for etanercept, 300 μg/ml for rituximab and tocilizumab, 1 μg/ml for MP, 0.8 μg/ml for cyclosporine A (CyA), 0.4 μg/ml for methotrexate, and 50 ng/ml for tofacitinib. CyA was chosen as a positive control drug with a known dose-dependent inhibitory effect on T-cell effector function and proliferation [13, 14]. After preincubation, samples were processed for cytokine secretion and proliferation analyses as described above. Because abatacept acts as a T-cell costimulation inhibitor by blocking the CD28-CD80/86 interaction, analyses of its effect on T-cell stimulation were performed in both the presence and absence of anti-CD28 antibody, which was routinely added together with CD49d to all stimulatory reactions (*see above*).

### Quantification of VZV-specific antibodies

VZV-specific antibodies were quantified using a commercial anti-IgG enzyme-linked immunosorbent assay (Euroimmun AG, Lübeck, Germany). IgG levels < 80 IU/L were scored negative, levels 80–110 IU/L were scored intermediate, and levels > 110 IU/L were scored positive according to the manufacturer’s instructions.

### Statistical analysis

Statistical data analysis was carried out using Prism version 5.03 software (GraphPad Software, La Jolla, CA, USA). Analyses of continuous variables (leukocyte count, percentage of lymphocytes, C-reactive protein [CRP], erythrocyte sedimentation rate [ESR], VZV- and SEB-reactive CD4 T-cell frequencies) were performed using the Mann-Whitney *U* test for two groups and the Kruskal-Wallis test (with Dunn’s posttest) for more than two groups. Differences in age, time since disease onset, Disease Activity Score 28-joint count (DAS28), and T-cell cytokine expression were analyzed using an unpaired *t* test for comparison between two groups and one-way analysis of variance (with Bonferroni posttest) for comparison of more than two groups. Comparison of categorical variables (erosive course, gender, history of herpes zoster) was performed using Fisher’s exact test and the χ^2^ test for two or more groups, respectively. Correlations were analyzed according to Spearman (rank-sum).

## Results

### Study population

VZV-specific immunity was analyzed in 98 samples of 90 patients with rheumatic diseases, including 70 patients (78 samples) with RA and 20 patients with different types of seronegative spondylarthritis (SpA, including 17 patients with PsA and 3 patients with AS). Samples from 39 age-matched immunocompetent individuals served as controls. Four patients (three RA, one SpA) showed VZV-specific IgG below the detection limit and were excluded from all subsequent analyses. Moreover, one sample of a patient who had an episode of zoster 1 month prior to sampling was excluded from major analyses. Their patient characteristics were not different from the remaining study participants, which are shown in Table 1. The three groups did not show any significant differences in gender, leukocyte counts, and percentages of lymphocytes. Likewise, time since onset of disease, DAS28, ESR, and the percentage of patients with an erosive course did not differ between patients with RA and patients with SpA, whereas CRP was significantly higher in patients with SpA (*p* = 0.03). In contrast to healthy control subjects, 12 of the 85 VZV-seropositive patients (11 RA, 1 SpA) reported a history of herpes zoster 8.2 ± 9.2 years ago (*p* = 0.03).

**Table 1:** Characteristics of the study population^a^

	Healthy control subjects	Patients with RA	Patients with seronegative SpA	*p* Value
Total number of tested samples	39	74	19^b^	–
Age, years, mean ± SD	58.6 ± 16.2	60.6 ± 13.0	51.4 ± 11.9	0.04^c,d^
Female sex, *n* (%)	22 (56.4)	56 (75.7)	11 (57.9)	0.07^e^
Leukocyte count median (IQR), cells/μl	7320 (3072) (*n* = 36)	7800 (4240) (*n* = 73)	7400 (4200) (*n* = 19)	0.42^f^
Lymphocytes median (IQR), %	26.0 (11.1) (*n* = 28)	23.0 (13.6) (*n* = 73)	26.0 (10.0) (*n* = 19)	0.12^f^
Time since disease onset, years, mean ± SD	–	8.0 ± 6.4 (*n* = 67)	7.9 ± 8.8 (*n* = 19)	0.93^g^
DAS28, mean ± SD	–	3.76 ± 1.27 (*n* = 50)	3.72 ± 1.48 (*n* = 3)	0.96^g^
CRP median (IQR), mg/L	–	1.9 (3.7) (*n* = 64)	4.3 (14.1) (*n* = 16)	0.03^h^
ESR median (IQR), mm/h	–	13.0 (18.5) (*n* = 52)	14.0 (18.0) (*n* = 15)	0.57^h^
Erosive course, %	–	40.0 (*n* = 60)	46.7 (*n* = 15)	0.77^i^
History of herpes zoster > 3 months ago, *n* (%)	0	11 (14.9)	1 (5.3)	0.03^e^

*Abbreviations: CRP* C-reactive protein, *DAS28* Disease Activity Score 28-joint count, *ESR* Erythrocyte sedimentation rate, *RA* Rheumatoid arthritis, *SpA* Seronegative spondylarthritis

The numbers in parentheses (n) refer to the samples for which the respective information was available

^a^VZV-IgG negative individuals (3 female RA patients (50, 58, and 77 years of age) and 1 male SpA patient (37 years of age), and one sample of an 80 years old female RA patient with an episode of herpes zoster 1 month before sampling were excluded (their leukocyte counts and clinical characteristics were not different from the remaining patients)

^b^Sixteen patients with psoriatic arthritis, three patients with ankylosing spondylitis

^c^In posttest, *p* < 0.05 only between RA and SpA

^d^One-way analysis of variance

^e^χ^2^ test

^f^Kruskal-Wallis test

^g^Unpaired *t* test

^h^Mann-Whitney *U* test

^i^Fisher’s exact test

### Lower percentages of VZV-specific and SEB-reactive CD4 T cells in patients with rheumatoid arthritis

**Fig. 1:**
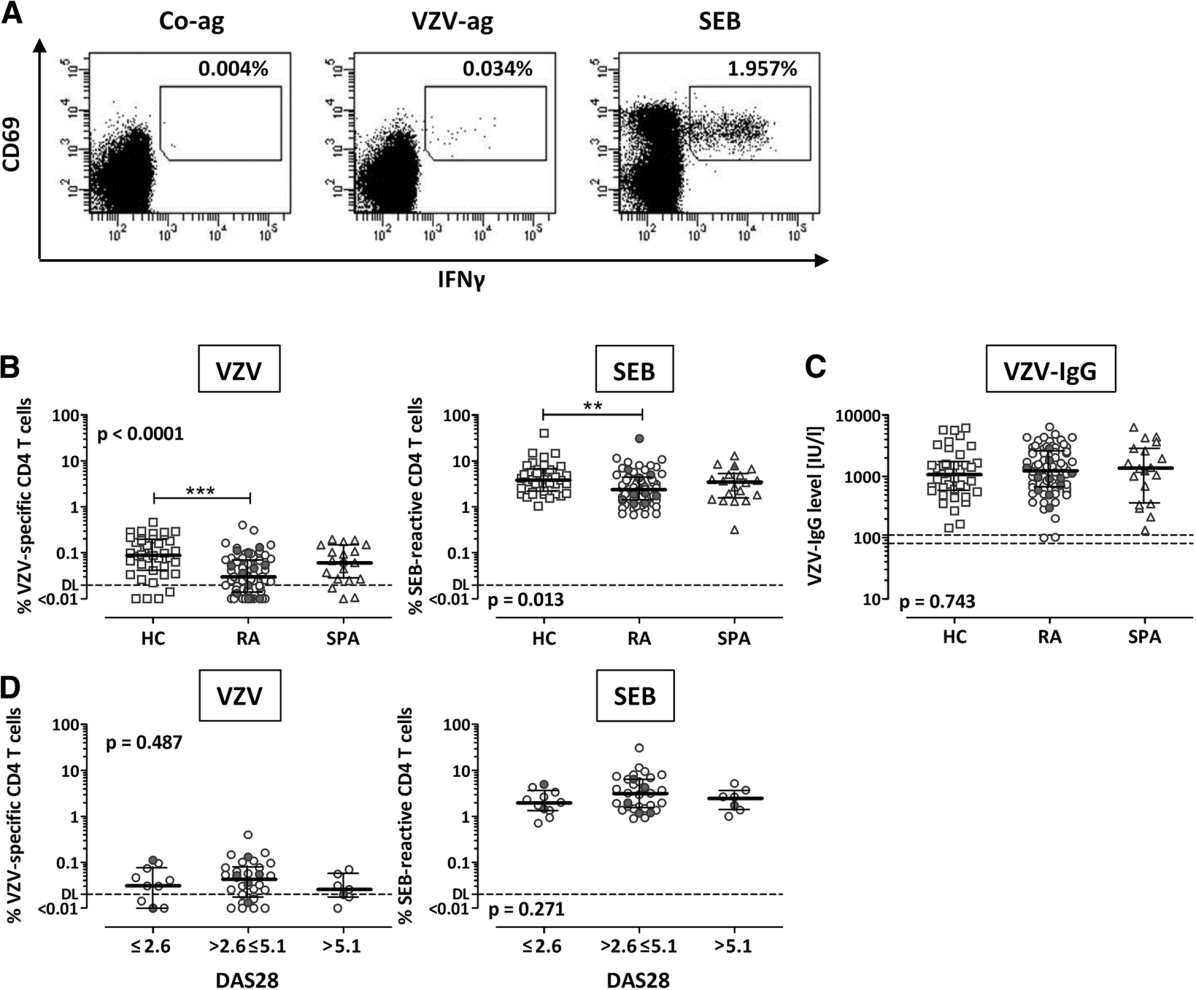
Varicella zoster virus (VZV)-specific and *Staphylococcus aureus* enterotoxin B (SEB)-reactive CD4 T cells show reduced frequencies in patients with rheumatoid arthritis (RA). Whole blood was stimulated with control antigen (Co-ag, negative control), VZV antigen (VZV-ag), and the polyclonal stimulus SEB, respectively, and reactive CD4 T cells (CD69^+^IFN-γ^+^) were analyzed using flow cytometry. **a** Typical dotplots of a 39-year-old patient with RA are shown. Numbers indicate the percentages of reactive CD4 T cells. **b** Frequencies of VZV-specific CD4 T cells corrected for the negative control (*left*) and of SEB-reactive CD4 T cells (*right*), comparing samples of healthy control subjects (HC, *n* = 39), patients with RA (*n* = 74) and seronegative spondylarthritis (SpA, *n* = 19). **c** Levels of VZV-specific antibodies (immunoglobulin G [IgG]) in HC, RA, and SpA determined by IgG enzyme-linked immunosorbent assay of plasma samples. **d** Stratification of VZV-specific (*left panel*) and SEB-reactive CD4 T-cell frequencies (*right panel*) of patients with RA according to disease activity (three DAS28 categories; ≤ 2.6, full remission, low disease activity, *n* = 11; > 2.6 to ≤ 5.1, moderate disease activity, *n* = 32; and > 5.1, high disease activity, *n* = 7). *Bars* indicate median values and IQRs; *dotted lines* depict the respective detection limits (DLs) as determined before (VZV 0.02%, SEB 0.05% [10]) or as indicated by the manufacturer (IgG). Gray symbols represent patients with known history of herpes zoster. Statistical analysis was performed using the Kruskal-Wallis test and Dunn’s posttest. Significant differences in posttest are marked by asterisks (*p < 0.05; ***p < 0.001)

To compare VZV-specific T-cell immunity in patients with RA, with SpA, and in healthy control subjects, whole-blood samples (*n* = 74, 19, and 39, respectively) were stimulated with VZV lysate and subsequently analyzed using flow cytometry. Stimulation with uninfected control lysate served as a negative control, and stimulation with SEB was performed for general assessment of polyclonally activated effector T cells. Reactive CD4 T cells were identified by coexpression of the activation marker CD69 and the cytokine IFN-γ after stimulation. Representative examples of flow cytometric dotplots are shown in Fig. 1a. CD4 T-cell frequencies in patients with RA and patients with SpA were lower than in control subjects, which held true for both VZV-specific (*p* < 0.0001) (Fig. 1b, left panel) and SEB-reactive CD4 T cells (*p* = 0.013) (Fig. 1b, right panel). In contrast, no differences were observed in VZV-specific IgG levels (Fig. 1c). As described before [10], there was an inverse correlation of VZV-specific T-cell levels with age (*r* = − 0.306, *p* = 0.008), which was not observed for SEB-reactive T cells (*r* = − 0.030, *p* = n.s.) (Additional file 1: Table S1). However, the lower percentage of reactive CD4 T cells in patients with RA was associated with neither disease activity (DAS28) (Fig. 1d) nor other clinical parameters (Additional file 1: Table S1). Likewise, no differences were found in VZV-specific and SEB-reactive CD4 T-cell frequencies between patients with and without a history of previous herpes zoster (gray and white symbols in Fig. 1). Interestingly, however, the patient who was excluded from the analyses owing to an episode of herpes zoster 1 month before sampling had a markedly higher percentage of VZV-specific CD4 T cells (0.396%) than in the time before zoster (0.010%; data not shown).

**Fig. 2:**
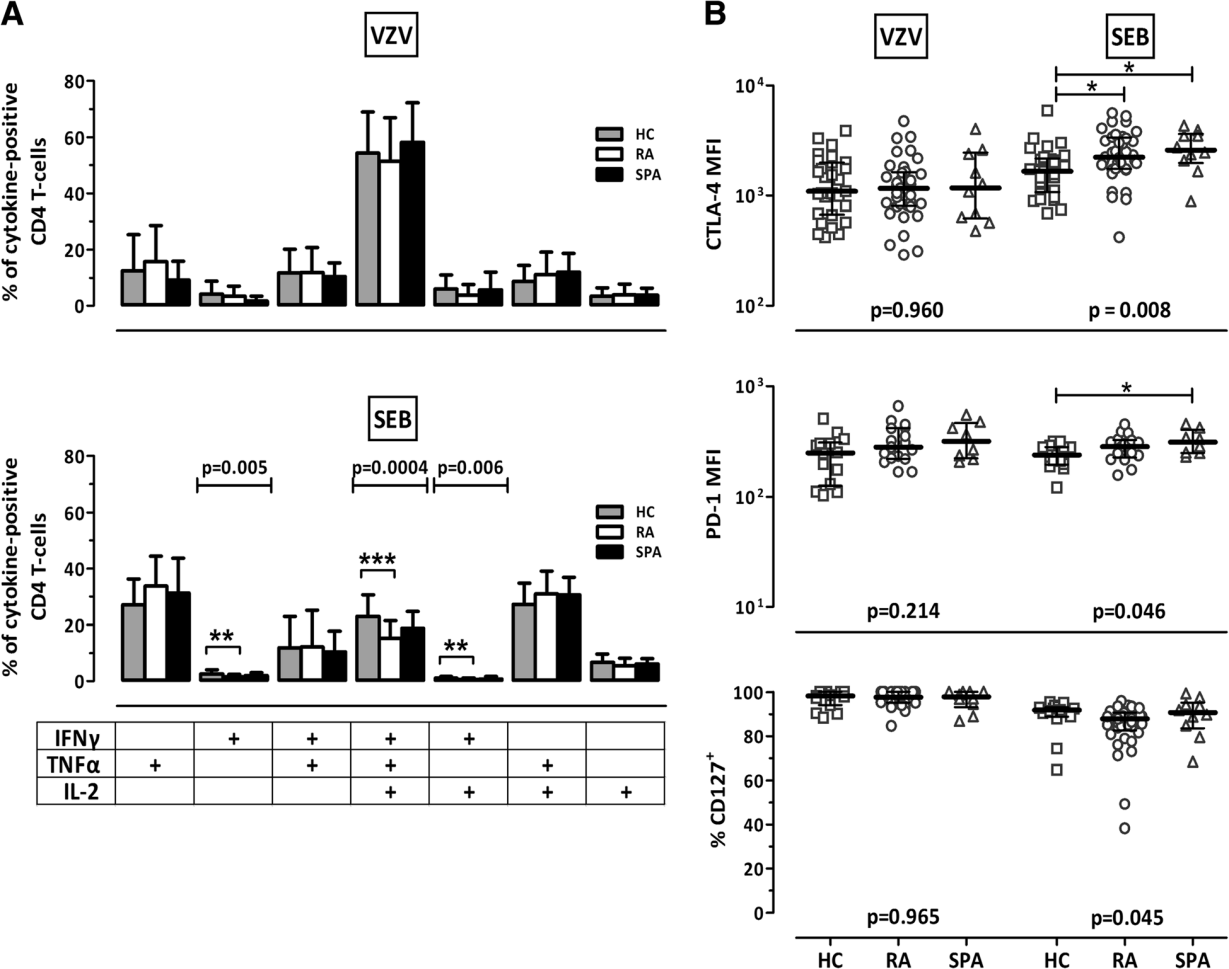
Moderate functional differences in polyclonally stimulated CD4 T cells of control subjects (HC) and patients with rheumatoid arthritis (RA). **a** Cytokine-expressing CD4 T cells of HC (*gray*), patients with RA (*white*), and patients with seronegative spondylarthritis (SpA; *black*) are divided according to their expression of interferon (IFN)-γ, interleukin (IL)-2, and tumor necrosis factor (TNF)-α after stimulation with varicella zoster virus antigen (VZV, *upper panel*) or *Staphylococcus aureus* enterotoxin B (SEB, *lower panel*). To ensure robust statistics, this analysis was restricted to all VZV-positive samples where at least 30 cytokine-producing CD4 T cells were detectable (29 samples for HC, 27 for RA, and 10 for SpA, respectively). Bars represent subpopulations of single-, double-, or triple-cytokine-producing cells among all VZV- or SEB-reactive CD4 T cells, including means and SDs. **b** Expression of the cytotoxic T-lymphocyte antigen 4 (CTLA-4), the programmed death 1 molecule (PD-1), and CD127 was analyzed on reactive (CD69^+^/IFN-γ^+^) CD4 T cells of HC, RA, and SpA. Only samples with at least 20 VZV-specific CD4 T cells were analyzed (*n* = 29 HC, 37 RA, and 10 SpA for CTLA-4; *n* = 14 HC, 17 RA, and 8 SpA for PD-1; and *n* = 15 HC, 37 RA, and 10 SpA for CD127). Statistical significance was assessed using one-way analysis of variance with Bonferroni posttest (**a**) or the Kruskal-Wallis test with Dunn’s posttest (**b**). Significant differences in posttests are marked by asterisks (*p < 0.05; **p < 0.01; ***p < 0.001)

To characterize functional and phenotypical properties of VZV- and SEB-reactive T cells, the expression profiles of the cytokines IFN-γ, IL-2, and TNF-α were analyzed, where a total of seven subpopulations expressing either one, two, or all three cytokines were considered. In addition, surface markers associated with functional anergy (CTLA-4, PD-1) and differentiation (CD127) were analyzed. Representative examples of the flow cytometric dotplots are depicted in Additional file 1: Figure S2. As shown in Fig. 2, VZV-specific CD4 T cells in patients did not exhibit any signs of functional impairment. In contrast, when compared with those of control subjects, SEB-reactive CD4 T cells of patients with RA showed lower percentages of cells expressing all three cytokines (*p* = 0.0004) or IFN-γ alone (*p* = 0.005) or in combination with IL-2 (*p* = 0.006) (Fig. 2a, lower panel). Likewise, SEB-reactive T cells from patients with RA and patients with SpA had higher expression levels of the anergy marker CTLA-4 (*p* < 0.05) (Fig. 2b, upper panel), whereas PD-1 expression was significantly higher in patients with SpA only (*p* < 0.05) (Fig. 2b, middle panel). In contrast, expression of the differentiation marker CD127 did not differ in any of the groups (Fig. 2b, lower panel).

Together, the results show significantly lower percentages of reactive CD4 T cells in patients with RA, but not in patients with SpA, when compared with control subjects (median threefold lower VZV-specific and median 1.7-fold lower SEB-reactive T cells). Functionality of VZV-specific CD4 T cells does not seem to be impaired, whereas polyclonally activated effector T cells showed minor but significant alterations in functionality in patients with RA and in patients with SpA.

### Influence of antirheumatic therapy on VZV-specific and SEB-reactive CD4 T cells in patients with RA

**Fig. 3:**
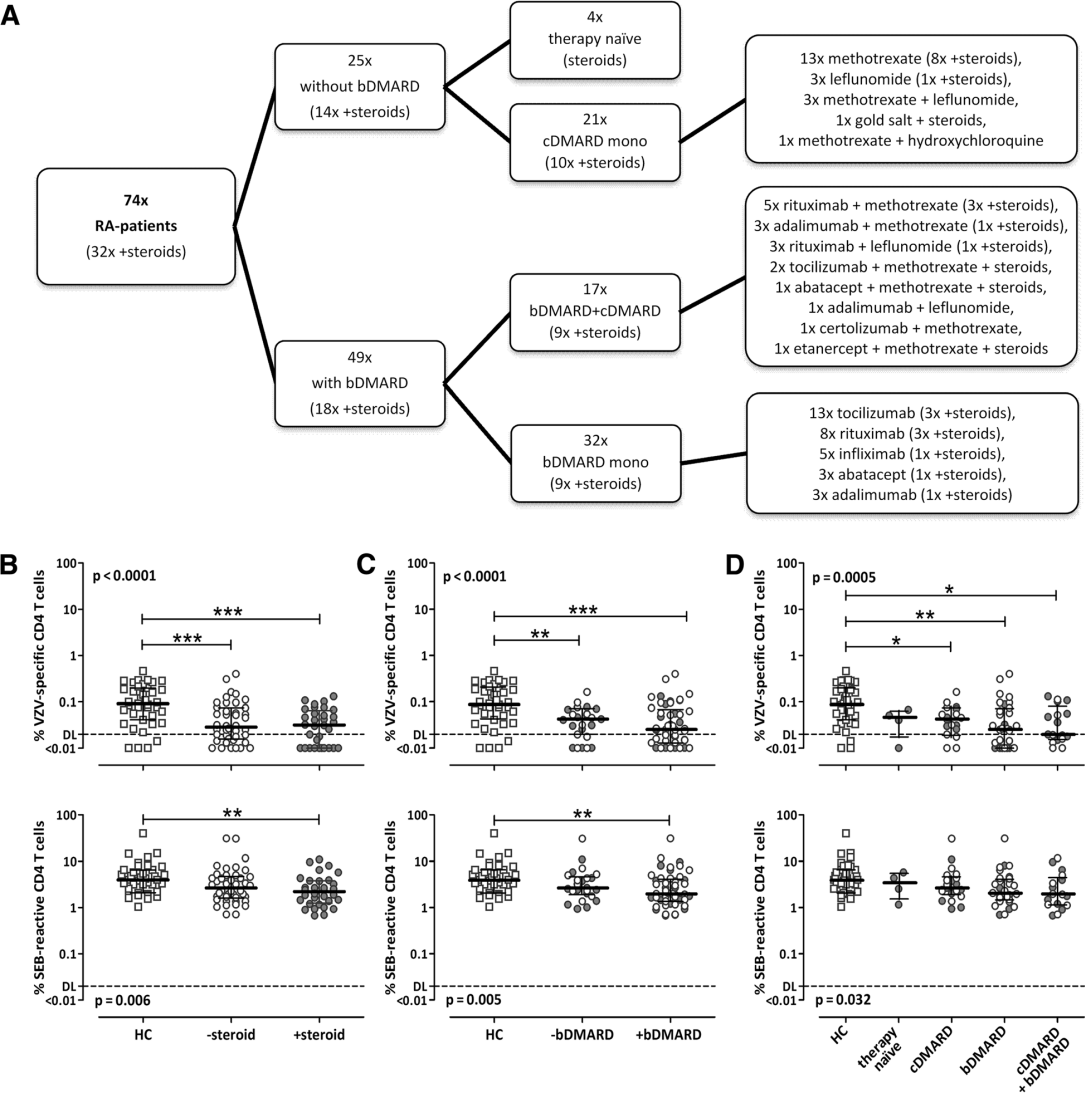
Antirheumatic therapy with biological disease-modifying antirheumatic drugs (bDMARDs) shows the strongest effect on varicella zoster virus (VZV)-specific T-cell frequencies in patients with rheumatoid arthritis (RA). **a** Flowchart gives an overview about the therapeutic regimens of the patients with RA, indicating the numbers of samples from patients with and without steroids, bDMARDs and conventional disease-modifying antirheumatic drugs (cDMARDs), including the detailed drug regimens. **b**–**d** Frequencies of VZV-specific (*upper panels*) and *Staphylococcus aureus* enterotoxin B (SEB)-reactive CD4 T cells (*lower panels*) of patients with RA were stratified according to therapy without (−) or with (+) steroids (**b**), bDMARDs independent of other medication (**c**), or bDMARDs and cDMARDs independent of steroids (**d**), and compared with respective frequencies of healthy control subjects (HC, *n* = 39). *Gray symbols* refer to patients receiving steroids as part of their medication. Statistical significance was assessed using the Kruskal-Wallis test with Dunn’s posttest (**b**–**d**). Significant differences in posttests are marked by asterisks (*p < 0.05, **p < 0.01, ***p < 0.001)

Because the immunological alterations in patients with rheumatic diseases were most pronounced in patients with RA, further analyses were restricted to this patient group. To evaluate potential effects of antirheumatic drugs on antigen-specific cellular immunity, patients were subdivided according to their specific antirheumatic medication (Fig. 3a), with or without corticosteroids, bDMARDs, cDMARDs, or combined cDMARDs/bDMARDs. Interestingly, VZV-specific T-cell levels were low in patients both with and without steroids, but they did not differ between the two groups (*p* < 0.001 when compared with control subjects, respectively) (Fig. 3b, upper panel). In contrast, the decrease in SEB-reactive T-cell levels was more pronounced for patients receiving steroids, indicating a potential role of this drug (Fig. 3b, lower panel). However, direct comparisons of VZV- and SEB-reactive cells between patients with and without steroids revealed no significant differences between the groups (*p* = 0.492 and *p* = 0.250, respectively). After stratifying the patients according to bDMARDs and nonbiological medications, significantly lower VZV-specific T-cell levels than in control subjects were found in both patients with and without bDMARDs, with lowest median frequencies in patients with bDMARDs (Fig. 3c, upper panel) (*p* < 0.001 and *p* < 0.01, respectively). Of note, the more pronounced impact of bDMARDs on VZV-specific T-cell frequencies was additionally evident by the fact that T-cell levels were low not only in patients who received bDMARDs alone but also in those who received them in combination with cDMARDs (*p* < 0.01 and *p* < 0.05, respectively) (Fig. 3d, upper panel). Likewise, SEB-reactive CD4 T-cell levels were lowest in patients with bDMARDs (*p* < 0.01) (Fig. 3c, lower panel), whereas no specific differences were found when stratified into treatment subgroups (Fig. 3d, lower panel). As highlighted by gray symbols, steroids did not have any influence on specific T-cell levels among the different treatment subgroups (Fig. 3c and d). Interestingly, there was also no correlation between the dosage of steroids and VZV-reactive (*r* = 0.098, *p* = n.s.) or SEB-reactive T-cell levels (*r* = − 0.191, *p* = n.s.) (Additional file 1: Table S1). Among cDMARDs, most patients received methotrexate (*n* = 30). Although cDMARDs decreased VZV-specific T-cell levels to some extent (Fig. 3d), T-cell levels did not differ between patients with and without methotrexate (Additional file 1: Figure S3). Taken together, among antirheumatic medications, bDMARDs had the most pronounced decreasing effect on VZV-specific T-cell frequencies, whereas SEB-reactive effector T cells seemed to be negatively influenced by glucocorticoids and/or bDMARDs.

### Differential impact of bDMARDs on T-cell immunity depending on their mechanism of action

**Fig. 4:**
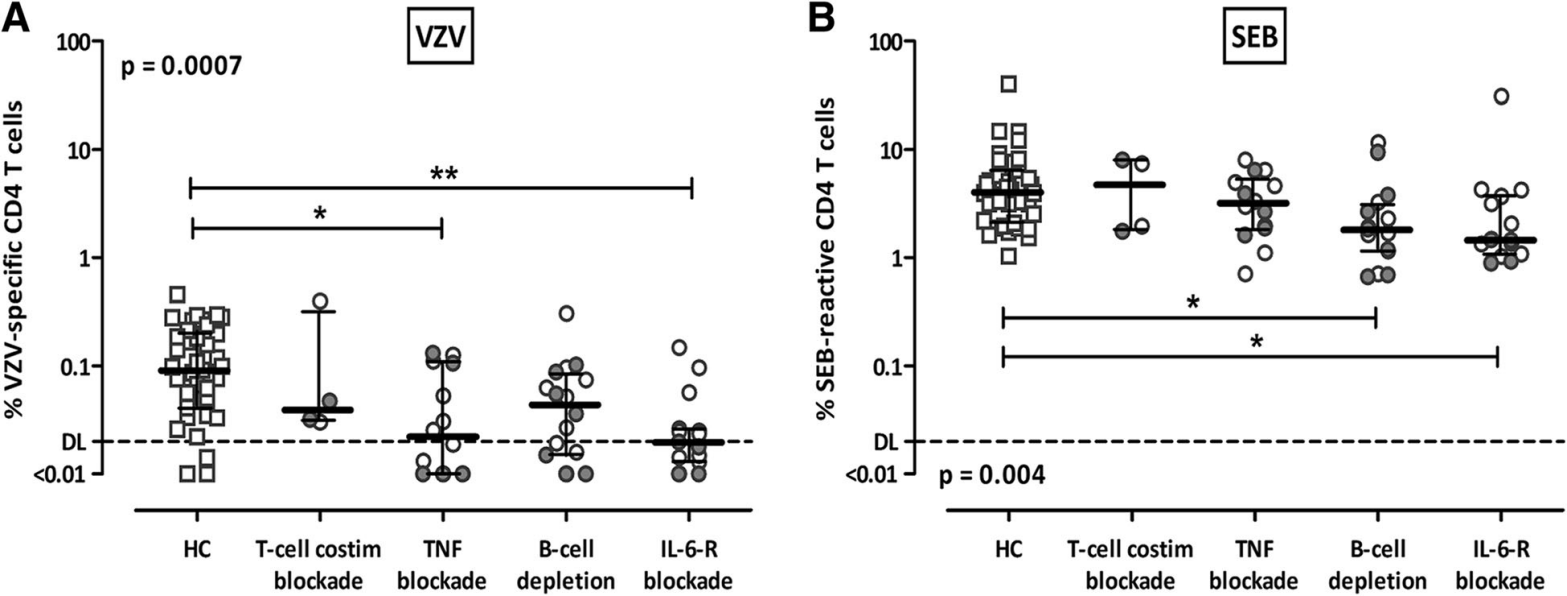
Specific biological disease-modifying antirheumatic drugs (bDMARDs) show differences in their effect on reactive CD4 T cells of patients with rheumatoid arthritis (RA). Scatterplots depict frequencies of varicella zoster virus (VZV)-specific **(a)** and *Staphylococcus aureus* enterotoxin B (SEB)-reactive **(b)** CD4 T cells of healthy control subjects (HC, *n* = 39) as well as patients with RA receiving biological disease-modifying bDMARDs, stratified according to the mechanism of action of the respective bDMARD (abatacept, T-cell costimulation blockade, *n* = 4; adalimumab, etanercept, infliximab, certolizumab, TNF blockade, *n* = 14; rituximab, B-cell depletion, *n* = 16; tocilizumab, interleukin [IL]-6R blockade, *n* = 15). *Bars* indicate median values and IQRs, *dotted lines* are detection limits for VZV-specific and SEB-reactive CD4 T cells as determined before (VZV 0.02%, SEB 0.05% [10]), respectively. *Gray symbols* refer to patients receiving steroids as part of their medication. Statistical differences were assessed using the Kruskal-Wallis test with Dunn’s posttest. Significant differences in posttests are marked by asterisks (*p < 0.05; **p < 0.01)

Because bDMARDs, which have the strongest influence on cellular immunity in patients with RA, differ in their mechanisms of action, we further analyzed their impact on VZV-specific and SEB-reactive T cells. Therefore, patients were divided into four groups receiving (1) the T-cell costimulation blocking agent abatacept; (2) TNF blocker adalimumab, etanercept, infliximab, or certolizumab; (3) the B-cell-depleting drug rituximab; or (4) the IL-6-receptor blocker tocilizumab. Comparison of the CD4 T-cell frequencies with those of healthy control subjects revealed a significant reduction, especially in patients receiving the IL-6 receptor blocker, both after VZV-specific (*p* < 0.01) (Fig. 4a) and after polyclonal stimulation (*p* < 0.05) (Fig. 4b). Three of four patients on abatacept had low levels of VZV-specific T cells, although statistical analysis of this effect is limited by the low number of patients available for recruitment. In addition, significant differences were detected for VZV-specific T cells of patients on TNF-blocking therapy (*p* < 0.05) and for SEB-reactive cells of patients receiving rituximab (*p* < 0.05) compared with healthy control subjects. Again, treatment with steroids did not have any influence on specific T-cell levels among subgroups of bDMARDs (gray symbols in Fig. 4).

To further evaluate the net effect of antirheumatic drugs independent of the disease background, whole-blood samples of three healthy control persons were supplemented in vitro with increasing concentrations of the different drugs and subsequently stimulated with VZV lysate or SEB as described before. Blood of nonimmunocompromised healthy control subjects was chosen to study direct effects of the drugs at defined dosages added in vitro without being confounded by effects of drugs that patients had received as part of their medication. The baseline VZV-specific T-cell levels of these individuals (0.061%, 0.086%, and 0.124%) were in the range of what is found for healthy control subjects (*see* Fig. 1b). Although CyA is not used in patients with RA, this drug served as an internal control for a known dose-dependent immunosuppressive effect on CD4 T-cell effector function in this in vitro system [13]. After 6 h of stimulation, coexpression of the activation marker CD69 with cytokine IFN-γ, IL-2, or TNF-α was determined by flow cytometry (Additional file 1: Figure S4A). Although the number of replicates was low (three VZV and three SEB stimulations), a pronounced dose-dependent decrease in cytokine-expressing T-cell frequencies was observed after incubation with the positive control drug CyA. To a lesser extent, this effect was also observed with MP. Interestingly, the only bDMARD that led to a similar dose-dependent decrease in VZV-specific and SEB-reactive T-cell frequencies was the TNF blocker adalimumab, but this inhibitory effect was restricted to the expression of TNF-α (Additional file 1: Figure S4A, lower panel), whereas there was no detectable effect on IFN-γ or IL-2 expression (Additional file 1: Figure S4A, upper or middle panel, respectively). All other tested drugs did not have any pronounced decreasing effect on cytokine induction in vitro. This also held true for methotrexate and the Janus kinase (JAK) inhibitor tofacitinib, which were tested in an independent series of five healthy individuals (Additional file 1: Figure S5A). When analyzing late cytokine expression (Additional file 1: Figure S4B and S5B) and proliferation (Additional file 1: Figure S4C and S5C) after 36 h of stimulation with SEB, CyA and MP had a clear dose-dependent inhibitory effect on both CD4 and CD8 T cells, whereas all other drugs had either no or no pronounced effect.

## Discussion

Patients with rheumatic diseases are at increased susceptibility for VZV reactivations. In line with this observation, the present study shows lower levels of VZV-specific T cells in patients with RA than in healthy control subjects. While this effect was not associated with disease severity, treatment regimens had a decreasing influence on cellular immunity, with regimens including bDMARDs showing the most pronounced effects.

Because VZV-specific CD4 T cells were shown to be essential for effective VZV control [8], the significantly lower frequencies of VZV-specific CD4 T cells in patients with RA may be the main explanation for their increased susceptibility to VZV reactivations. The pronounced role of the cellular arm of adaptive immunity is supported by the fact that VZV IgG levels were not concomitantly impaired. This is consistent with observations in patients with SLE who showed a significant reduction in VZV-specific T-cell frequencies, even in the presence of increased VZV IgG levels [9]. This lends support to the assumption that a poor VZV-specific cellular but not humoral immunity may be the main contributor to increased zoster incidence in patients with RA. In our study, the median percentage of VZV-specific T cells in patients with SpA was also lower than in control subjects, but this effect was less pronounced than that in patients with RA, which may indicate that the extent of immune impairment may depend on the entity of rheumatic disease. Indeed, the incidence of herpes zoster in different groups of patients with autoimmune and inflammatory diseases ranges from 6.8 per 1000 person-years in patients with gout to 19.9 per 1000 person-years in patients with SLE [4]. Interestingly, consistent with effects on VZV-specific CD4 T cells in our study, the incidence rate in patients with RA was higher than that of patients with PsA and AS [4].

We have previously shown that patients with herpes zoster [10] and VZV-associated meningitis [15] show increased levels of VZV-specific T cells with limited ability to produce cytokines and with increased expression levels of markers of functional anergy such as CTLA-4 or PD-1. These parameters were typical characteristics of VZV-specific T cells not only in immunocompetent symptomatic patients but also in patients with immunodeficiency, including RA, which normalized after resolution of zoster within approximately 3 months to levels comparable to those of individuals without history of zoster [10]. Although patients with a history of zoster were included in the present study, their zoster episode was several years ago, and none of the patients showed any evidence of herpes zoster or other clinical signs of VZV reactivation at the time of analysis. As with our findings in immunocompetent control subjects [10], VZV-specific T-cell levels in patients with a history of herpes zoster did not differ from those of patients who never experienced a VZV reactivation. Despite absence of clinical symptoms, quantitative differences in VZV-specific T-cell levels were observed between patients and control subjects, whereas functionality and cell surface expression of anergy markers were largely unaffected. When analyzing SEB-reactive T cells as a surrogate for T cells with other specificities, those also showed decreased levels. Moreover, there was also evidence for a reduced percentage of multifunctional T cells and increased expression of CTLA-4 and PD-1, which were similar to those in asymptomatic transplant recipients receiving immunosuppressive therapy [10, 16–18]. Notably, however, the detected changes were less pronounced than those found in patients with active infection. These data are consistent with recent findings that bulk CD4 T cells from patients with RA and SpA were shown to have slightly elevated expression levels of CTLA-4 or PD-1 [19]. Together, this may reflect increased susceptibility for infections and/or may be the result of a history of more frequent infections of patients with rheumatic diseases than in immunologically healthy persons.

The decrease in T-cell levels may be a direct effect of immunosuppressive and immunomodulatory drugs and further influenced by the underlying disease. Because patients typically received immunosuppressive drug combinations at the time of recruitment, the net influence of individual drugs is difficult to assess. In line with a clear dose-dependent association between the use of corticosteroids and zoster risk [20–23], our in vitro experiments in which MP was supplemented in concentrations equivalent to 4 and 400 mg/d indicate an involvement of steroids in reducing effector T-cell frequencies. The observation that differences in patients with RA with and without steroids were only significant for polyclonally stimulated T cells may be due to the fact that steroid dosage in our patients varied from as little as 1.5 to up to 40 mg/d with only four patients receiving ≥ 10 mg/d, and most patients received additional immunomodulatory drugs such as bDMARDs, which may have unmasked the effect of an individual drug. Thus, differences may result from the fact that the in vitro experiments analyzed potential direct effects of each individual immunosuppressive drug on T-cell effector function, whereas T-cell analyses in patients in vivo are influenced by the combined action of several immunosuppressive drugs that most patients received simultaneously.

Up to now, several studies have reported an increased incidence and severity of VZV reactivations in patients with rheumatic disease receiving therapy with bDMARDs [20, 24–26]. In line with that, we observed significantly reduced cellular immune responses in patients receiving therapy with different bDMARDs. Among those, the IL-6 receptor blocker and TNF antagonists had the most pronounced effect on VZV-specific CD4 T-cell frequencies in patients with RA. TNF antagonists may have both direct and indirect effects on VZV replication. Given the role of TNF-α as an effective inhibitor of VZV replication [27], a causative role of TNF antagonist therapy for an increased incidence of VZV reactivations is conceivable. Moreover, stimulation of blood samples from healthy control subjects in the presence of the TNF blocker adalimumab dose-dependently impaired TNF-α induction in CD4 T cells while not affecting IFN-γ or IL-2, which indicates an immediate drug effect on effector T-cell function independent of the underlying disease. In patients on TNF antagonist therapy, a decrease in TNF-α signaling may negatively affect nuclear factor (NF)-κB activation, which may have a direct inhibitory effect on immune cells. TNF antagonists may also negatively affect VZV-infected cells in addition to the gene product of VZV-ORF61, which is capable of inhibiting NF-κB activation owing to its E3 ubiquitin ligase activity [28]. Together, both processes may synergistically favor VZV reactivation events. As with effects of TNF antagonists on the VZV-specific immune response, patients receiving the IL-6 receptor blocker tocilizumab showed significantly lower VZV-specific CD4 T-cell frequencies than control subjects. However, because frequencies of SEB-reactive T cells were also reduced, the inhibitory effect may be less specific. In patients with SLE, treatment with tocilizumab led to a decrease in the percentage of HLA-DR^+^ activated T cells [29]. Given that IL-6 inhibits regulatory T-cell (Treg) development by inducing a shift towards T-helper type 17 cell development, blocking of IL-6 signal transduction by tocilizumab induces higher amounts of Tregs, which might result in suppression of cytokine expression in effector T cells [30, 31]. We finally also found that polyclonal T-cell responses were significantly reduced in patients receiving rituximab, whereas its effect on VZV-specific CD4 T cells was less pronounced. Although this drug primarily targets B cells via binding to CD20, adverse effects on T cells are also known [32]. Apart from reduced T-cell responses by missing antigen presentation of B cells, rituximab may directly influence cytokine expression of T cells by binding to CD20-positive T cells [33]. If so, it is tempting to speculate whether VZV-specific T cells are largely CD20-negative and thus less susceptible to rituximab treatment. More recently, treatment regimens including JAK inhibitors such as tofacitinib and baricitinib were also shown to be associated with a high rate of herpes zoster [34, 35]. Although we did not find any pronounced effect of tofacitinib on T-cell effector function in vitro, studying the effect of these novel drugs on VZV-specific cellular immunity in vivo may be an interesting area of future research to better understand increased susceptibility for VZV reactivations.

Our study may be limited by sample size and lack of follow-up data to study quantitative levels of specific T-cell immunity in relation to clinical reactivation events. Therefore, future longitudinal studies in larger cohorts of patients with RA should address whether a progressive decrease in specific immunity and functional impairment may be used as a predictor for VZV reactivations. This type of study with a larger sample size will also allow simultaneous characterization of VZV-specific immunity in association with the frequency of VZV infections on therapy with different bDMARDs.

## Conclusions

Patients with RA show a decreased level of antigen-specific CD4 T cells, with VZV-specific T cells being particularly influenced by bDMARDs such as TNF antagonists and IL-6 receptor blockers, which may account for an increased incidence of VZV reactivations. Our findings are relevant for clinical practice because they emphasize the importance of preventive measures such as zoster vaccinations. Moreover, together with our findings on phenotypical and functional changes of specific immunity in patients with active VZV infection [10, 15], this knowledge may be useful for cellular diagnostics to assess individual immunocompetence toward this clinically relevant pathogen. Finally, this study may be extended to study VZV-specific immunocompetence in patients with novel synthetic disease-modifying antirheumatic drugs known to be associated with increased risk of herpes zoster.

## Additional file

Additional file 1: **Table S1.** Correlation between VZV-specific or SEB-reactive CD4 T-cell levels and demographic and clinical parameters in patients with RA. **Figure S1.** VZV-specific CD4 T cells are detectable after varicella vaccination. **Figure S2.** Representative examples of flow cytometric analyses. **Figure S3.** Analysis of the influence of methotrexate on VZV-specific and SEB-reactive CD4 T cells. **Figure S4.** In vitro effect of antirheumatic drugs on cytokine expression and proliferation of reactive T cells. **Figure S5.** In vitro effect of tofacitinib and methotrexate on cytokine expression and proliferation of reactive T cells. (DOC 2282 kb)

## Abbreviations

AS: Ankylosing spondylitis
BrdU: Bromodeoxyuridine
cDMARD/bDMARD: Conventional/biologic disease-modifying antirheumatic drug
CRP: C-reactive protein
CTLA-4: Cytotoxic T-lymphocyte antigen 4
CyA: Cyclosporine A
DMARD: Disease-modifying antirheumatic drug
ESR: Erythrocyte sedimentation rate
IFN-γ: Interferon-γ
IgG: Immunoglobulin G
IL: Interleukin
JAK: Janus kinase
MP: Methylprednisolone
NF-κB: Nuclear factor-κB
PD-1: Programmed death 1 molecule
PsA: Psoriatic arthritis
RA: Rheumatoid arthritis
SEB: *Staphylococcus aureus* enterotoxin B
SLE: Systemic lupus erythematosus
SpA: Seronegative spondylarthritis
TNF-α: Tumor necrosis factor–α
Treg: Regulatory T cell
VZV: Varicella zoster virus
