# Supplementary material for: VZV-specific T-cell levels in patients with rheumatic diseases are reduced and differentially influenced by antirheumatic drugs
Source: Arthritis Res Ther. 2018 Nov 9;20:252. doi: 10.1186/s13075-018-1742-5 (PMC6235212; doi:10.1186/s13075-018-1742-5)
Supplement: Supplementary file 1 — Table S1. Correlation between VZV-specific or SEB-reactive CD4 T-cell levels and demographic and clinical parameters in patients with RA. Figure S1. VZV-specific CD4 T cells are detectable after varicella vaccination. Figure S2. Representative examples of flow cytometric analyses. Figure S3. Analysis of the influence of methotrexate on VZV-specific and SEB-reactive CD4 T cells. Figure S4. In vitro effect of antirheumatic drugs on cytokine expression and proliferation of reactive T cells. Figure S5. In vitro effect of tofacitinib and methotrexate on cytokine expression and proliferation of reactive T cells. (DOC 2282 kb) [file 13075_2018_1742_MOESM1_ESM.doc]

# Additional file 1

# VZV-specific T-cell levels in patients with rheumatic diseases are reduced and differentially influenced by antirheumatic drugs

David Schub, Gunter Assmann, Urban Sester, Martina Sester, and Tina Schmidt

Supporting documents include

- 1 Table S1
- 5 Figures S1–S5.

# Table S1

# Table S1: Correlation between VZV-specific or SEB-reactive CD4 T-cell levels and demographic and clinical parameters in patients with rheumatoid arthritis

| Correlation | VZV-specific CD4 T cells [%] | SEB-reactive CD4 T cells [%] |
| --- | --- | --- |
| Age [years] | **r=-0.306 p=0.008** | r=-0.030 p=n.s. |
| Leukocyte counts [cells/µL] | r=0.089  p=n.s. | r=-0.016 p=n.s. |
| Percentage of lymphocytes | r=0.074 p=n.s. | r=-0.045 p=n.s. |
| Time since disease onset [years] | r=-0.168 p=n.s. | r=-0.015 p=n.s. |
| DAS28 | r=0.237 p=n.s. | r=0.187 p=n.s. |
| CRP [mg/L] | r=-0.066 p=n.s. | r=-0.056 p=n.s. |
| ESR [mm/h] | r=-0.221 p=n.s. | r=-0.009 p=n.s. |
| Steroid dosage [mg/d] | r=0.098 p=n.s. | r=-0.191 p=n.s |

r-values were calculated according to Spearman.

# Figure S1


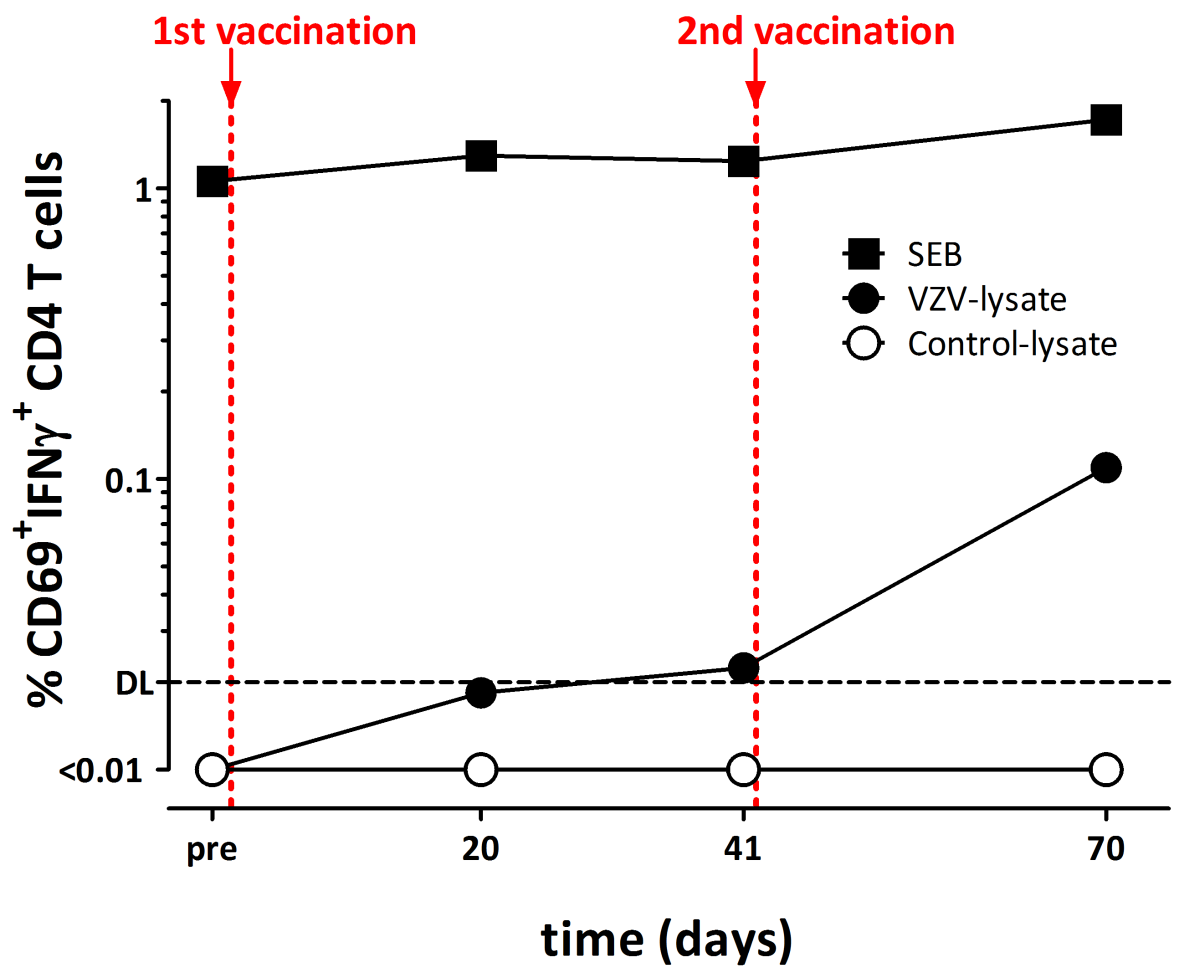


**Figure S1: VZV-specific CD4 T cells are detectable after varicella vaccination.** VZV-specific CD4 T cells were quantified after stimulation with the VZV-lysate in a 12-year old boy before and after two vaccinations with the varicella vaccine (after written informed consent by the parents). Stimulation with control-lysate was carried out as negative control, stimulation with *Staphylococcus aureus* Enterotoxin B (SEB) served as positive control. VZV-specific T-cell levels increase after the first vaccination and are boosted further after the second vaccination, whereas SEB-reactive T-cell levels remain constant, and uninfected control lysate did not elicit any T-cell response. DL, detection limit

# Figure S2

#
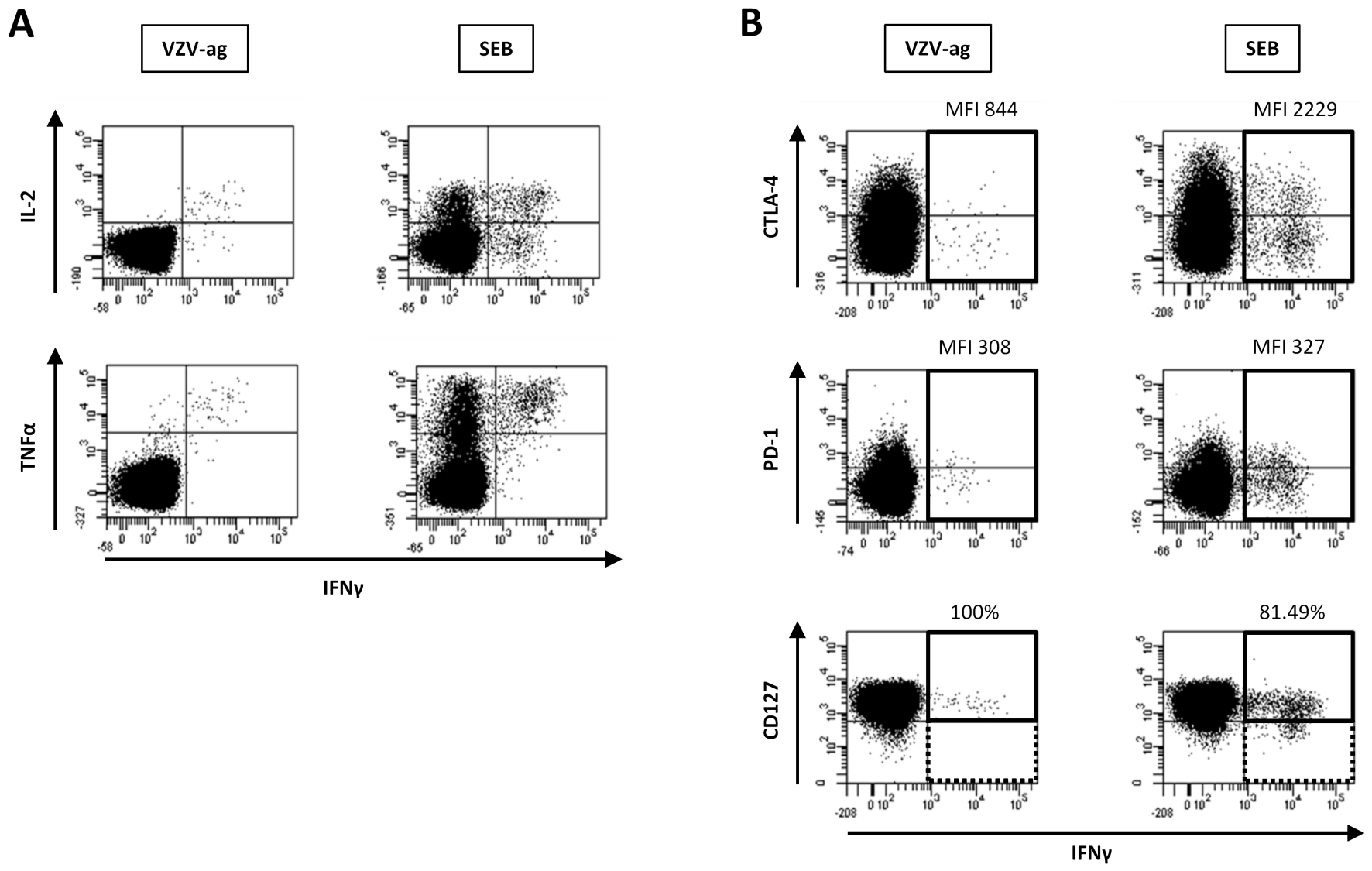


**Figure S2: Representative examples of flow-cytometric analyses.** Expression of **(A)** the cytokines IFNγ, IL-2 and TNFα and **(B)** of the cytotoxic T-lymphocyte antigen 4 (CTLA-4), the programmed death (PD)-1 molecule, and CD127 was analyzed on reactive (CD69^+^/IFNγ^+^) CD4 T cells. Shown are typical dotplots of CD4 T cells of a 49 year old female patient with rheumatoid arthritis after stimulation with VZV-antigen (left) or *Staphylococcus aureus* enterotoxin B (SEB, right), respectively. Numbers in (B) indicate the mean fluorescence intensity (MFI) for CTLA-4 and PD-1 expression, respectively, on IFNγ-expressing CD4 T-cells, or the percentage of CD127-positive cells among all IFNγ-expressing CD4 T-cells.

# Figure S3


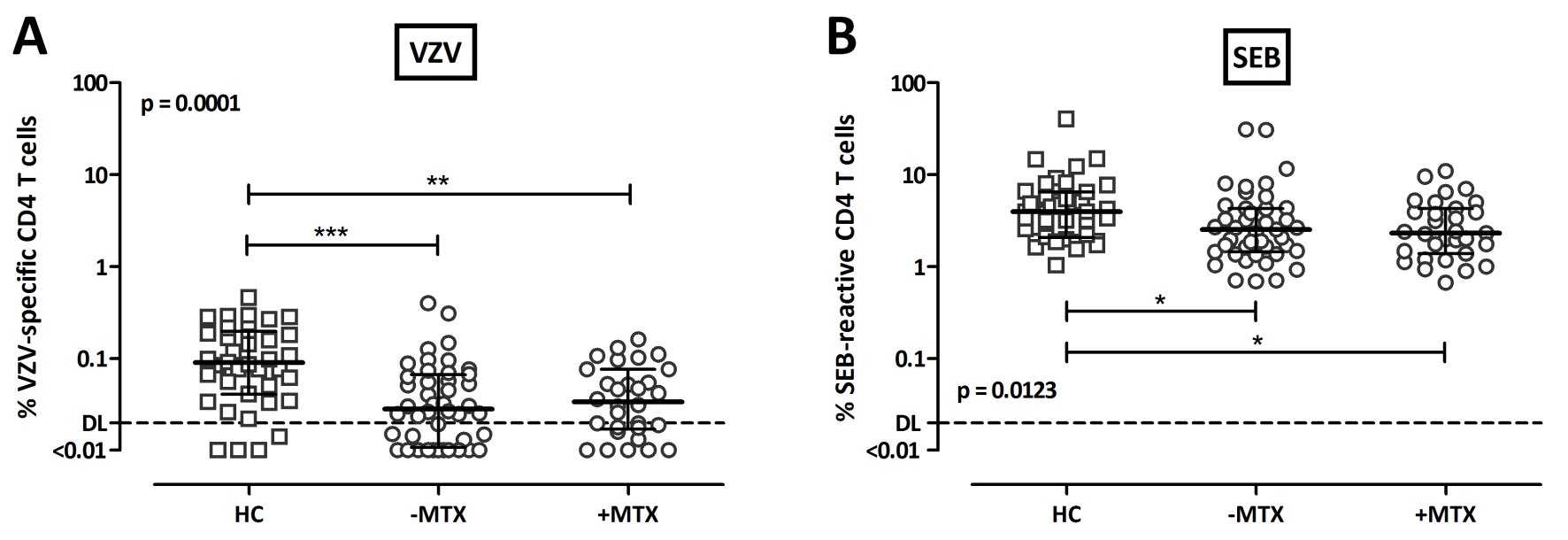


**Figure S3: Analysis of the influence of methotrexate on VZV-specific and SEB-reactive CD4 T cells.** Frequencies of **(A)** VZV-specific and **(B)** SEB-reactive CD4 T cells of RA patients were stratified according to therapy without methotrexate (-MTX, n=44) or with methotrexate (+MTX, n=30) and compared to respective frequencies of healthy controls (HC, n=39). Statistical significance was assessed using Kruskal-Wallis test with Dunn’s post-test. Significant differences in post-tests are marked by asterisks (* for p<0.05, ** for p<0.01, *** for p<0.001).

# Figure S4


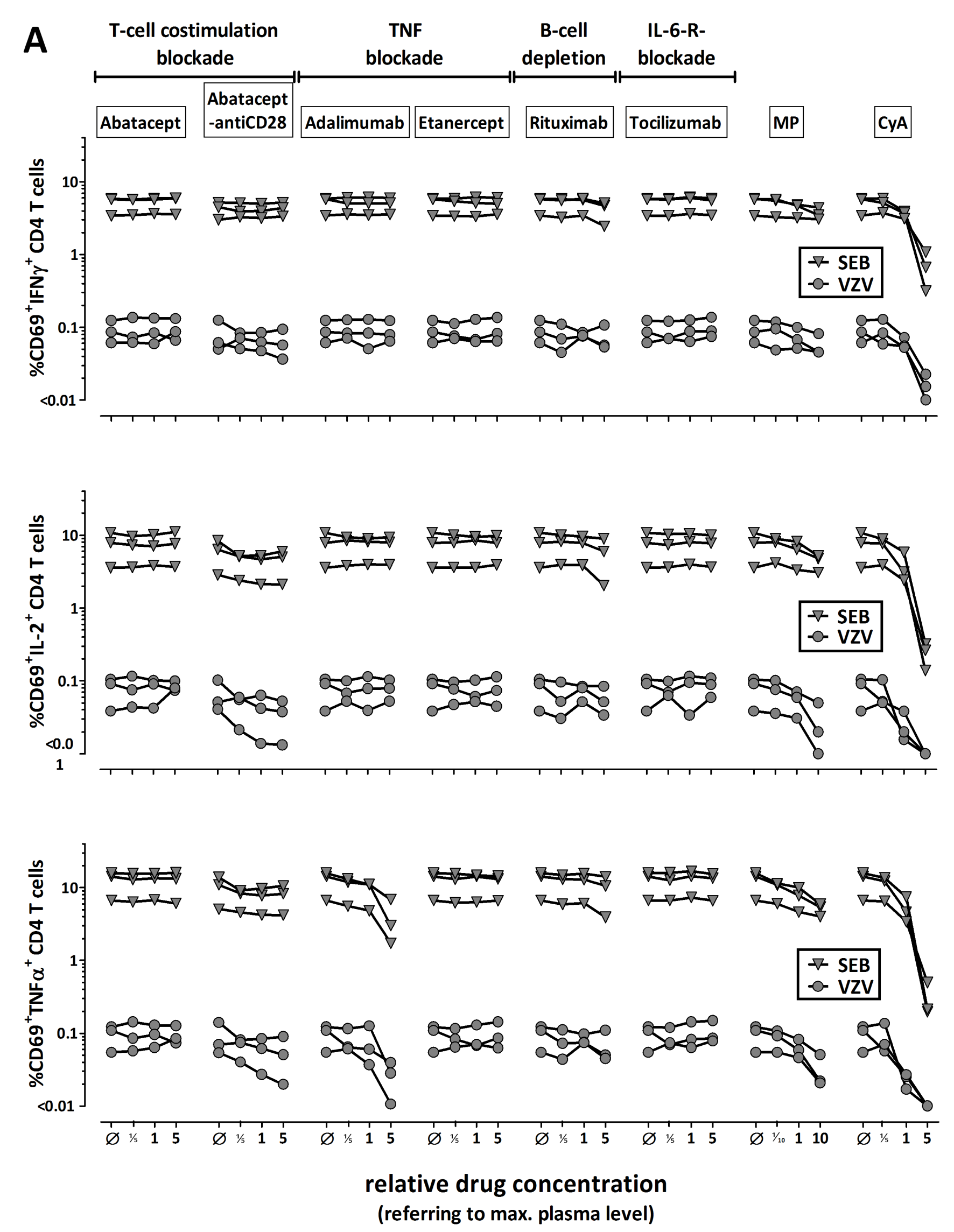


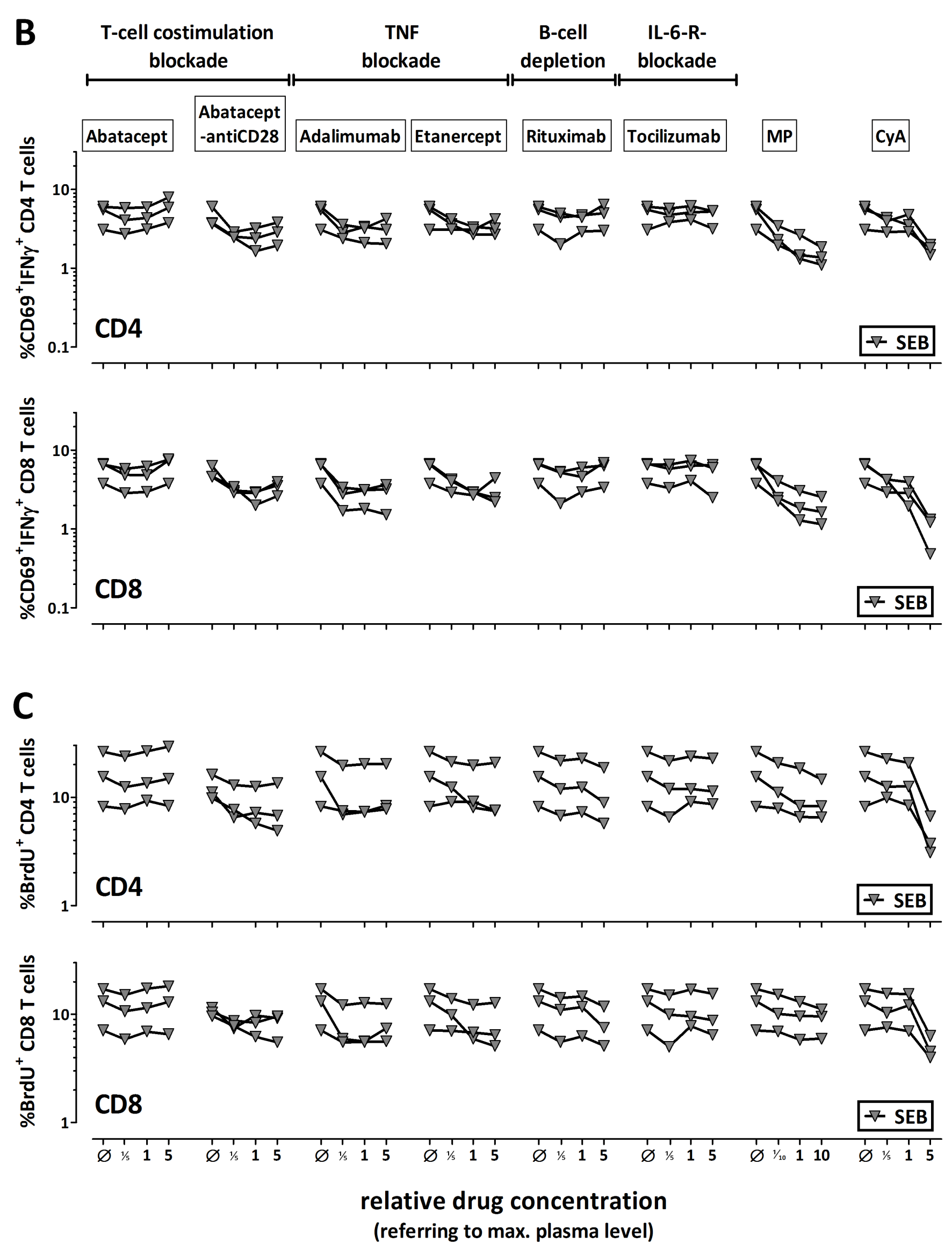


**Figure S4: In-vitro effect of antirheumatic drugs on cytokine expression and proliferation of reactive T cells.** Blood samples of 3 healthy controls were supplemented with titrated amounts of different bDMARDs in vitro for 4 hours, subsequently stimulated with VZV-antigen or SEB, respectively, as indicated and flow-cytometrically analyzed after additional **(A)** 6 hours for assessment of early co-expression of CD69 and interferon (IFN) γ (upper panel), interleukin (IL) 2 (middle panel) or tumor necrosis factor (TNF) α (lower panel) respectively of CD4 T cells, or **(B)** 36 hours for assessment of late co-expression of CD69 and IFNγ of CD4 T cells (upper panel) and CD8 T cells (lower panel) respectively, or **(C)** 36 hours and addition of bromodeoxyuridine (BrdU) for assessment of proliferation of CD4 T cells (upper panel) and CD8 T cells (lower panel) respectively. As abatacept acts as T-cell costimulation inhibitor by blocking the CD28-CD80/86 interaction, analyses of its effect on T-cell stimulation were performed once with and once without anti-CD28 antibody which is routinely added together with CD49d in all other samples to optimize the stimulatory effect. VZV-specific CD4 T-cell frequencies were rather low after 6h of stimulation, where maximum cytokine expression signals are expected. Therefore, analyses in (B) and (C) were restricted to SEB-reactive T cells. The three individuals were 26, 27, and 34 years of age (3 females). Titrations represent ranges of drug concentrations, where “1” refers to the estimated maximum plasma level that is reached in patients after a single drug uptake (150µg/ml for abatacept, 100µg/ml for adalimumab, 2.5µg/ml for etanercept, 300µg/ml for rituximab and tocilizumab, 0.8µg/ml for cyclosporine A and 1µg/ml for methylprednisolone); Ø = no drug administration.

# Figure S5


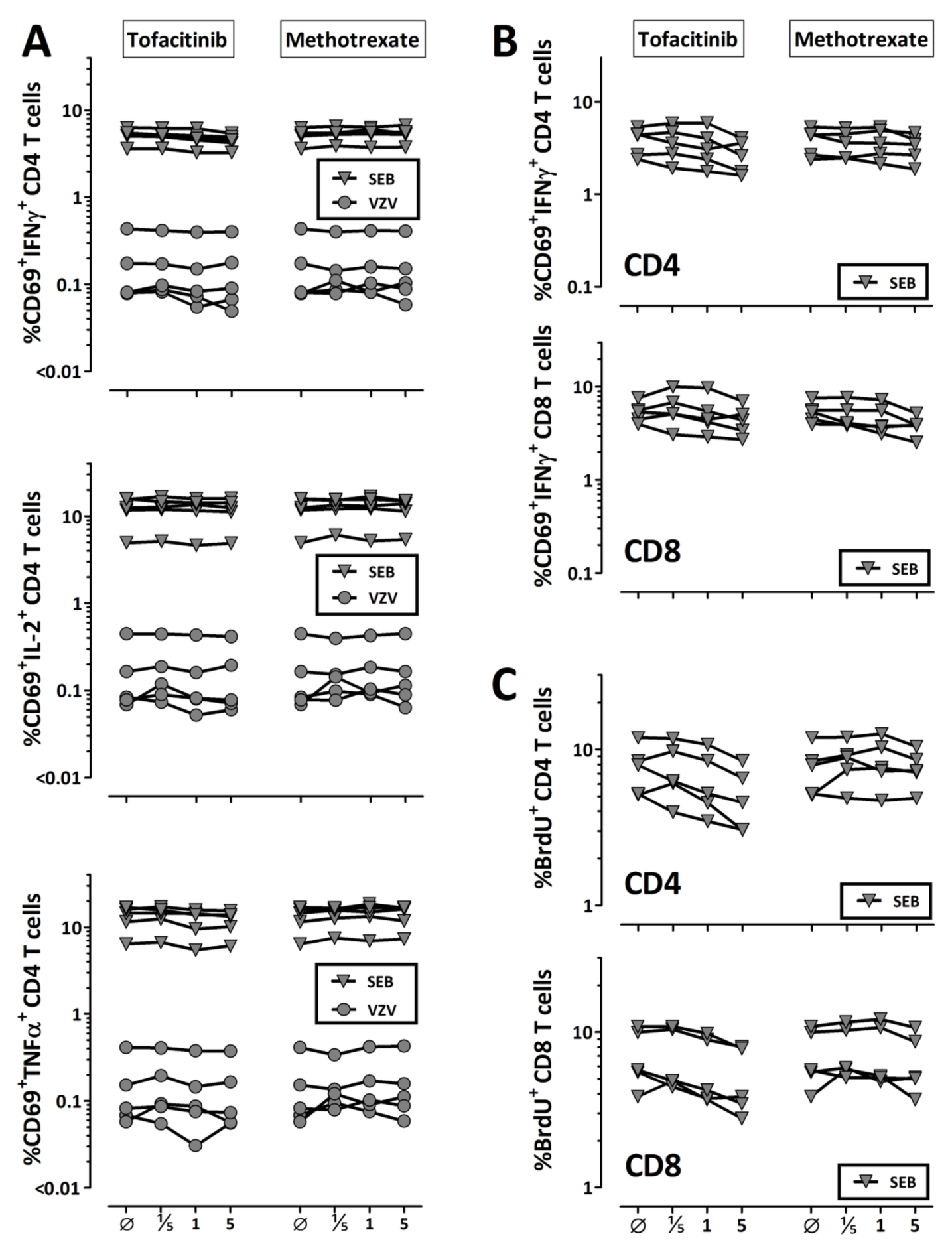


**Figure S5: In-vitro effect of** **tofacitinib and methotrexate on cytokine expression and proliferation of reactive T cells.** Blood samples of 5 healthy controls were supplemented with titrated amounts of tofacitinib and methotrexate in vitro for 4 hours, subsequently stimulated with VZV-antigen or SEB, respectively, as indicated and flow-cytometrically analyzed after additional **(A)** 6 hours for assessment of early co-expression of CD69 and interferon (IFN) γ (upper panel), interleukin (IL) 2 (middle panel) or tumor necrosis factor (TNF) α (lower panel) respectively of CD4 T cells, or **(B)** 36 hours for assessment of late co-expression of CD69 and IFNγ of CD4 T cells (upper panel) and CD8 T cells (lower panel) respectively, or **(C)** 36 hours and addition of bromodeoxyuridine (BrdU) for assessment of proliferation of CD4 T cells (upper panel) and CD8 T cells (lower panel) respectively. VZV-specific CD4 T-cell frequencies were rather low after 6h of stimulation, where maximum cytokine expression signals are expected. Therefore, analyses in (B) and (C) were restricted to SEB-reactive T cells. The five female individuals were 35.0±5.4 years of age. Titrations represent ranges of drug concentrations, where “1” refers to the estimated maximum plasma level that is reached in patients after a single drug uptake (50ng/ml for tofacitinib, 0.4µg/ml for methotrexate); Ø = no drug administration.
